# Rapid dissolving microneedle patch integrated with benidipine-loaded nanotransfersomes for transdermal drug delivery: optimization, characterizations, and preclinical bioavailability assessment

**DOI:** 10.1007/s13346-025-01976-9

**Published:** 2025-10-15

**Authors:** Khater AL-Japairai, Samah Hamed Almurisi, Fawaz Alheibshy, Nadiya Abdul-Halim, Syed Mahmood

**Affiliations:** 1 Department of Pharmaceutical Engineering, Faculty of Chemical and Process Engineering Technology, Universiti Malaysia Pahang Al-Sultan Abdullah, 26300 Gambang, Malaysia; 2 Department of Pharmaceutical Technology, School of Pharmacy, International Medical University (IMU), 57000 Kuala Lumpur, Malaysia; 3 Department of Pharmaceutics, College of Pharmacy, University of Hail, 2240 Hail, Saudi Arabia; 4 Department of Pharmaceutical Technology, Faculty of Pharmacy, Universiti Malaya, 50603 Kuala Lumpur, Malaysia; 5 Research Centre for Biopharmaceuticals and Advanced Therapeutics (UBAT), Department of Pharmacology, Faculty of Medicine, Universiti Malaya, Universiti Malaya, 50603 Kuala Lumpur, Malaysia; 6 Centre of Advanced Materials (CAM), Faculty of Engineering, Universiti Malaya, 50603 Kuala Lumpur, Malaysia; 7 Faculty of Pharmaceutical Sciences, Chulalongkorn University, Pathum Wan, 10330 Bangkok Thailand

**Keywords:** Benidipine, Transfersomes, Dissolving Microneedles, Transdermal Drug Delivery, Bioavailability

## Abstract

Benidipine hydrochloride (BEN) is widely prescribed for managing hypertension; however, its clinical efficacy is limited by poor oral bioavailability. This study introduces an innovative delivery approach, incorporating BEN-loaded nanotransfersomes (BEN-TF) into a rapid dissolving microneedles (DMNs) patch for transdermal administration. The nanotransfersomal formulation was optimised via a Box–Behnken design following preparation using a thin-film hydration method. The optimised formulation exhibited favourable characteristics, including a vesicle size of 124.9 ± 1.49 nm, high entrapment efficiency (98.12 ± 0.18%), and transdermal flux of 9.74 ± 0.53 μg/cm^2^/hr. DSC, ATR-FTIR, and XRD analyses confirmed the amorphous state of BEN. Imaging via FESEM and HRTEM demonstrated spherical, uniform nanosized vesicles. Confocal microscopy revealed deep skin penetration. The integration of BEN-TF into DMNs (BEN-TF-DMNs) resulted in efficient skin insertion, rapid dissolution, and good mechanical strength. Ex-vivo results indicated superior permeation compared to BEN-TF or BEN-DMNs alone, while the in-vivo study confirmed improved bioavailability versus both oral tablets and BEN-DMNs. This hybrid delivery platform offers a promising strategy for improving the systemic delivery of BEN.

**Graphical abstract:**

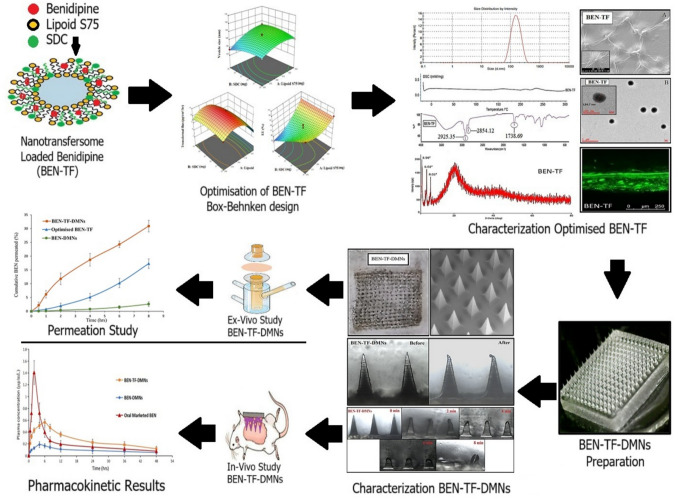

## Introduction

Hypertension is typically defined by a sustained increase in systolic blood pressure to 140 mm Hg or higher, and/or diastolic pressure of at least 90 mm Hg [1]. Data from Malaysia’s National Health and Morbidity Survey (NHMS) indicate that approximately 30.3% of adults aged 18 and above are affected, with the incidence rising by 6.7% annually [2]. Benidipine hydrochloride (BEN), a synthetic dihydropyridine derivative, is frequently prescribed by physicians to treat coexisting conditions such as angina pectoris and hypertension. It belongs to the calcium channel blocker class and is available in tablet form, typically administered at a dose of 2–4 mg per day, which may be gradually increased up to 8 mg depending on the patient's needs. Due to extensive hepatic first-pass metabolism, the oral bioavailability of BEN has been reported to be as low as approximately 23–30% [3]. Its low bioavailability and high first-pass metabolism necessitate frequent administration of high daily oral doses, which increases the risk of adverse effects and reduces patient compliance.

Lipid vesicles have effectively evolved as vehicles for transdermal drug delivery. The vesicular systems reduce medical expenses by increasing the bioavailability of medications with low solubility [4]. Additionally, lipid vesicles offer superior biocompatibility and safety, prolong the drug’s half-life, regulate the release of drug molecules, and protect the drug from physiological degradation [5]. Transfersomes are flexible bilayer vesicles formed by the combination of phospholipids and edge activator surfactants [6]. Several advantages of transfersomes over conventional liposomes have been reported [7–9]; transfersomes are highly elastic and amenable to skin penetration due to their deformability, which allows them to cross narrow pores in the skin. Furthermore, due to their flexible nature, they can effectively carry large amounts of lipophilic drugs (~ 90%). Additionally, transfersomes are stable, biocompatible, and biodegradable. They are capable of penetrating deep below the epidermal layers and are suitable for delivering both hydrophilic and lipophilic drugs. Additionally, they can transport both low- and high-molecular-weight drugs across the skin. Examples of drugs compatible with transfersome delivery include analgesics, anaesthetics, sex hormones, insulin, corticosteroids, anticancer agents, gap junction protein, and albumin [10]. Dissolving microneedles (DMNs), on the other hand, are arrays of micron-long needles made of biodegradable, water-soluble polymers. The efficiency of transdermal delivery is enhanced by the painless and minimally invasive insertion of DMNs into the epidermis, creating hundreds of microscopic channels in the skin [11, 12]. As DMNs are composed of water-soluble polymers, they offer several advantages, including rapid dissolution and short application times, which improve patient compliance and minimise skin tissue damage [13].

Despite decades of research, chemical penetration enhancers such as lipid vesicles have shown limited success in enhancing the transdermal transport of small molecules. In contrast, external devices have been more effective in improving the efficiency of transdermal drug delivery [14]. As a result, combining lipid vesicles with DMNs represents a significant advancement in the development of transdermal patches. DMNs can serve as an effective means of delivering the drug deeper into the dermis and subsequently into the systemic circulation, thereby enhancing bioavailability. Accordingly, this study explores a transdermal drug delivery strategy that combines lipid vesicles, serving as a chemical penetration enhancer, with DMNs, acting as a physical enhancer, to effectively overcome the skin barrier. It is hypothesised that this combined system will synergistically improve the transdermal delivery and systemic bioavailability of benidipine hydrochloride (BEN). Previous attempts to improve its absorption, including solid dispersions and nanosuspensions, have shown only limited success [15]. The transdermal route for BEN delivery has received little attention. To date, one study reported the development of matrix-type patches using Methocel E15LV and Eudragit RL100, which demonstrated promising in-vitro diffusion across goat skin; however, no in-vivo evaluation was conducted [16]. Our group has also previously investigated the incorporation of BEN-loaded ethanolic lipid nanovescicles (ethosomes) into dissolving microneedles, which showed a marked improvement in transdermal delivery [17]. These limited reports underscore the novelty of the present work, which advances beyond conventional patch-based systems by integrating BEN-loaded nanotransfersomes into DMNs. Compared to previously reported approaches aimed at improving the bioavailability of various drugs that employed either lipid vesicles or microneedles alone, this dual system achieved superior entrapment efficiency and significantly enhanced in vivo bioavailability.

## Materials and methods

Benidipine hydrochloride (BEN) of pharmaceutical-grade purity (≥ 98%) and paclitaxel (≥ 99%) were sourced from Binzhou Neophr Pharmaceutical Co., Ltd. (China). Soy phospholipid with 70% phosphatidylcholine content (Lipoid S75) was obtained from Lipoid GmbH, Switzerland. Sodium deoxycholate (SDC), hyaluronic acid (HA; molecular weight 10 kDa), methanol, chloroform, and ethanol (HPLC-grade) were purchased from Sigma-Aldrich (USA) and Fisher Scientific (Malaysia). Polydimethylsiloxane (PDMS) micromoulds were supplied by Micropoint Technologies (Singapore).

### Formulation of benidipine-loaded transfersomes (BEN-TF)

Transfersomes containing benidipine hydrochloride (BEN) were prepared using a thin-film rotary evaporation method with slight modifications [18, 19]. BEN (10 mg), Lipoid S75 (250–350 mg), and SDC (25–35 mg) were co-dissolved in a 2:1 v/v methanol–chloroform mixture within a round-bottom flask. The organic solvents were removed using a rotary evaporator (IKA RV-8, USA) at 40 °C and 100 rpm under reduced pressure to form a lipid film. This film was vacuum-dried overnight to eliminate residual solvents, then rehydrated with 10 ml of ultrapure water (pH 6.67) at 42 ± 2 °C using an orbital shaker for one hour. The dispersion was left at ambient temperature for two hours to promote vesicle formation. To achieve nanoscale size, the hydrated vesicles were sonicated using a probe sonicator (Qsonica, USA) at 20% amplitude for 25–45 s, then filtered through a 0.45 µm nylon membrane. Formulations were stored at 4 °C until further use.

### Optimisation of benidipine loaded transfersome formulations (BEN-TF)

A Box–Behnken design (BBD) with three independent variables: phospholipid concentration (A), surfactant amount (B), and sonication time was employed to optimise the BEN-TF formulation using Design Expert software (v13.0.5.0). The dependent variables were vesicle size (Y₁), entrapment efficiency (Y₂), and transdermal flux (Y₃). The design included 17 experimental runs with five replicates at the centre point to ensure reproducibility. Relationships between variables were analysed using 3D response surface plots [20]. All experiments were conducted in triplicate, and results expressed as mean ± standard deviation Table 1.

**Table 1:** Variables of box-Behnken design for BEN-TF formulations

Factors	Levels		
Independent variables	Low (−1)	Medium (0)	High (+ 1)
A = Amount of phospholipid: Lipoid S75 (mg)	250	300	350
B = Amount of surfactant: Sodium deoxycholate (SDC) (mg)	25	30	35
C = Sonication time (sec)	25	35	45
Dependent variables	Goal		
Y1 = Vesicles size (nm)	Minimize		
Y2 = Entrapment efficiency (EE%)	Maximize		
Y3 = Transdermal flux (μg/cm^2^/hr)	Maximize		

### Characterization of optimised benidipine loaded transfersome (BEN-TF)

#### Vesicle size, polydispersity index (PDI), and zeta potential measurement

The average vesicle diameter and PDI were measured using dynamic light scattering (DLS) with a Zetasizer Nano S90 (Malvern Instruments, UK) at 25 ± 5 °C and 173° scattering angle [21]. The zeta potential was also assessed using the Zetasizer Nano S90 (Malvern Instruments Ltd., UK) [22]. Samples were appropriately diluted in ultrapure water before analysis.

#### Entrapment efficiency (EE%) study

The entrapment efficiency (EE%) of the BEN-TF formulations was determined by centrifugation to separate the vesicles from the unentrapped drug using a high-speed refrigerated centrifuge (CR22N, Hitachi, Koki Co., Ltd., Japan) at 20,000 rpm for 20 min at 4 °C [23]. The free drug present in the supernatant was quantified using a validated HPLC method, based on the calibration curve (Y = 27.294X + 21.669; R^2^ = 0.9998) [17].The entrapment efficiency was calculated using the following equation [24]:

1 $${\mathrm{EE}({\%})}=\frac{\mathrm{A}_1-\mathrm{A}_2}{\mathrm{A}_1}\times 100$$

where A₁ is the initial amount of BEN, A₂ is the amount of BEN detected in the supernatant, and A₁ − A₂ represents the amount of BEN entrapped in the vesicles.

#### BEN content in optimised BEN-TF formulation

The BEN content in the optimised BEN-TF formulation was determined by dissolving 1 ml of the formulation (containing 1 mg/ml of BEN) in 10 ml of methanol using a vortex mixer (V-1 Plus, Biosan, Latvia) for 5 min. The mixture was then centrifuged at 4,000 rpm for 20 min at 25 °C to separate the methanol-dissolved BEN (supernatant) from the remaining vesicles [25]. The amount of BEN in the supernatant was subsequently quantified using a validated HPLC method. The drug content (DC%) was calculated using the following equation (Eq. 2):

2 $$\text{Drug content}=\frac{BEN\ quantified\ in\ optimised\ formulation}{Theoretical\ amount\ of\ BEN\ used}\times 100$$

#### Ex-vivo skin permeation study of BEN-TF formulations

The permeation study was conducted using rat abdominal skin mounted on a Franz diffusion cell (receptor volume: 12 ml; area: 1.5 cm^2^). The receptor chamber contained PBS (pH 7.4) and ethanol (60:40 v/v), maintained at 37 ± 2 °C with continuous stirring. Samples were collected at defined intervals and replaced with fresh buffer. The collected samples were analysed using a validated HPLC method. Control groups included BEN in hydroethanolic solution (BEN-HE) and conventional liposomes (BEN-CL). Permeation parameters (flux, Jss, and permeability coefficient, Kp) were calculated using following equations:

3 $$\mathrm{Kp}=\frac{Jss}{C0}$$

4 $$\text{Enhancement ratio}=\frac{Steady\ state\ flux\ of\ formulation }{Steady\ state\ flux\ of\ control}$$

#### Compatibility study using differential scanning calorimetry (DSC) and attenuated total reflectance spectroscopy (ATR-FTIR)

The thermal behaviour functional groups of pure BEN powder, Lipoid S75, sodium deoxycholate (SDC), and the freeze-dried optimised BEN-TF formulation was evaluated using DSC (Netzsch, Germany) and FTIR spectrometer (Thermo Fisher Scientific, Waltham, MA, USA). For DSC analysis, approximately 5–8 mg of each sample was weighed and sealed in an aluminium crucible with a pinhole in the lid. The samples were subjected to a controlled temperature range from − 20 °C to 300 °C under a constant nitrogen flow of 20 ml/min, with a heating rate of 10 °C/min [26]. DSC thermograms were recorded using Stare software (version 9.10). For FTIR, approximately 25–50 mg of each sample was firmly pressed against the ATR diamond crystal. The samples were scanned across the infrared range of 4000–400 cm⁻^1^ under a force of less than 90 units [27]. The resulting spectra were analysed and compared visually using OMNIC™ software (Thermo Electron Corporation, Madison, WI, USA), focusing on characteristic peaks corresponding to the functional groups of interest.

#### X-ray diffraction (XRD)

The crystallinity of BEN in the optimised freeze-dried BEN-TF formulation was assessed using X ray diffraction (XRD). In addition, individual excipients were also analysed. Diffraction patterns were obtained using an X ray Diffractometer (Rigaku Ultima IV, Japan). The diffraction angles (2θ) and corresponding intensity (counts) were recorded and compared to evaluate the crystalline or amorphous nature of BEN in both the pure drug and the optimised formulation [28].

#### Field emission scanning electron microscopy (FESEM) and high-resolution transmission electron microscopy (HRTEM)

The structural morphology and size of the optimised BEN-TF formulation was evaluated using FESEM (JEOL, JSM-7800F, Akishima, Japan) and HRTEM (JEOL, JEM-2100F). For FESEM, a droplet of the sample was placed onto a glass coverslip and allowed to dry. The dried specimen was then coated with a thin layer of platinum to enhance conductivity. Imaging was performed at an accelerating voltage of 5.0 kV, with magnifications of 3000 × [29]. For HRTEM, a negative staining technique was applied to enhance vesicle contrast. A few small droplets of the prepared vesicle suspension were placed onto a copper grid coated with a thin film. The vesicles were stained with 1% (w/v) phosphotungstic acid at pH 7.1. The sample was then dried on the grid and imaged at magnifications of 40 k ×, using an accelerating voltage of 200 kV [30].

#### Confocal laser scanning microscopy (CLSM)

CLSM was used to evaluate the skin deposition and distribution of the optimised BEN-TF formulation in comparison with a conventional liposomal formulation (BEN-CL). Following an ex-vivo skin permeation study using Franz diffusion cells for 8 h, the treated skin samples were thoroughly rinsed with ultrapure water and sectioned vertically into slices of 8–12 µm thickness. The skin sections were examined at the Faculty of Pharmacy, UiTM, using a Leica TCS SPE confocal microscope (Germany). The depth of vesicle penetration and fluorescence intensity within different skin layers were analysed along the Z-axis using Leica Advanced Fluorescence (LAS SF) software.

### Integration of optimised BEN-TF into DMNs (BEN-TF-DMNs)

The optimised BEN-TF formulation was incorporated into dissolving microneedles (DMNs) using a micromould casting technique. The moulds employed in this research are made of polydimethylsiloxane (PDMS), which consist of a 15 × 15 array with needle heights of 600 μm, a width at base of 200 μm and an 8 mm × 8 mm patch size, which were purchased from Micropoint Technologies Pte. Ltd. (Singapore). To accommodate the limited drug-loading capacity of microneedles, the BEN-TF formulation was first concentrated by reducing the volume via rotary evaporation. Initially, 10 ml of BEN-TF (1 mg/ml) was condensed to 1 ml, yielding a final concentration of 10 mg/ml. This concentrated formulation was blended in a 1:1 ratio with an 80% (w/v) hyaluronic acid (HA) solution, producing a final HA concentration of 40%. The resulting viscous mixture was degassed for 10 min to eliminate air bubbles and then cast into PDMS micromoulds. The moulds were centrifuged at 4000 rpm for 60 min to facilitate cavity filling, followed by air drying for 10 min at room temperature. Subsequently, a 50% (w/v) HA solution was applied as a backing layer. Another centrifugation step was carried out under identical conditions, and the complete DMN arrays were dried at 30 °C for 12 h. The final microneedle patches were carefully removed using medical-grade adhesive tape pre-mounted with a backing film. For comparison, control DMNs containing BEN in hydroethanolic solution (BEN-DMNs) were prepared using the same procedure, substituting the concentrated BEN-TF with 1 ml of BEN solution (10 mg/ml).

#### Morphology of BEN-TF-DMNs

The microneedle arrays were examined under a digital microscope (U500, Shenzhen, China) and an inverted optical microscope (Evos XL Life, Thermo Fisher Scientific, USA) for visual inspection of shape and uniformity. Dimensional measurements were carried out using ImageJ^®^ software. A scanning electron microscope (SEM; Carl Zeiss AG-EVO^®^ 50 Series, Germany) was used to analyse surface features and needle fidelity [31].

#### Mechanical strength of HA-DMNs and BEN-TF-DMNs

To determine mechanical robustness, a CT3 texture analyser (Brookfield, USA) was employed in compression mode [32, 33]. The microneedle arrays were pressed vertically against a flat aluminium surface with a force of 32 N for 30 s at a speed of 0.03 mm/s. The reduction in microneedle height was measured pre- and post-compression under an inverted microscope, and the percentage height reduction was computed using ImageJ^®^[34].

#### Insertion capability of BEN-TF-DMNs

Insertion efficiency was tested in vitro using Parafilm M^®^ (Bemis Company, Belgium) folded into eight layers, with a total thickness of 1 mm. Microneedles were applied using a texture analyser at 32 N for 30 s. The number of perforations per layer was counted under a digital microscope to determine insertion depth. The insertion depth was evaluated by plotting the percentage of holes created at each layer depth [34, 35]. For ex vivo validation, BEN-TF-DMNs were applied to excised rat abdominal skin. Following application and removal, skin sites were examined microscopically to assess the percentage of successfully inserted microneedles [36].

#### Dissolution of BEN-TF-DMNs in rat skin

Microneedle dissolution in skin was assessed over time. The BEN-TF-DMNs were applied to excised rat skin using a spring-loaded applicator (1.6 N force). Arrays were removed at pre-determined intervals (1 to 8 min), and microneedle integrity was observed via inverted microscopy. Needle length reduction was quantified using ImageJ^®^ to estimate the extent of dissolution [37].

#### Determination of BEN content in BEN-TF-DMNs

To quantify BEN in the needle tips, both BEN-TF-DMNs and control BEN-DMNs were dissolved in 3 ml hydroethanolic solution and vortexed for 10 min. Samples were centrifuged (4000 rpm, 10 min), and the supernatant was filtered (0.45 µm nylon) before HPLC analysis.

#### Ex-vivo skin permeation study of BEN-TF-DMNs

The skin permeation potential of BEN-TF-DMNs was compared against two control groups: optimised BEN-TF and BEN-DMNs. The experimental setup used Franz diffusion cell integrated. Rat abdominal skin was fixed between the donor and receptor compartments, with the stratum corneum side facing the donor chamber. The application of DMNs was performed using a calibrated spring applicator delivering a consistent impact force of 1.6 N. To maintain uniform skin–formulation contact, a 5 g stainless steel weight was gently placed atop each microneedle patch. The receptor chamber was filled with a hydroalcoholic buffer (PBS, pH 7.4: ethanol, 60:40 v/v), maintained at 37 ± 2 °C and stirred at 250 rpm. At specified time points (0.5, 1, 2, 4, 6, and 8 h), 0.3 ml samples were withdrawn and replaced immediately with fresh medium to sustain sink conditions. Samples were analysed using a validated HPLC method (Y = 29.948X + 0.15; R^2^ = 0.9996), and cumulative permeation profiles were plotted over time. The enhancement ratio (ER) and permeability coefficient (Kp) were calculated as described in the Compatibility study using differential scanning calorimetry (DSC) and attenuated total reflectance spectroscopy (ATR-FTIR) section.

#### Pharmacokinetic study

The ethical approval for animal study was obtained from the Institutional Animal Care and Use Committee (IACUC) of Universiti Malaysia Pahang (UMP), Approval No: UMPIACUC/2021/02. Nine male Sprague–Dawley rats (180 ± 20 g, 6–8 weeks old) were acquired from Sapphire Enterprise (Selangor) and housed in standard laboratory cages (three rats per cage) under controlled conditions (25 ± 3 °C, 12-h light/dark cycle, 50 ± 10% humidity). Animals were acclimatised for one week and fasted overnight before the study, but water was provided for them. A 10 cm^2^ area on the dorsal side of each rat was shaved 24 h before transdermal application to ensure full exposure. The site was inspected for irritation before the experiment [38]. The rats were randomly assigned into three groups (n = 3 per group) as follows: oral control: BEN marketed tablet (Coniel^®^), transdermal control: BEN-DMNs, and test group: BEN-TF-DMNs. Each group received a dose equivalent to 0.822 mg/kg of BEN via the respective administration route. On the day of the experiment, the rats were anaesthetised via intraperitoneal injection of pentobarbital (50 mg/kg in a volume of 0.83 ml/kg). Blood samples (0.5 ml) were collected from the retro-orbital vein at predetermined times (0.5, 1, 2, 4, 6, 8, 12, 24, 36, and 48 h) using EDTA tubes as anticoagulants. The collected samples were gently mixed and centrifuged at 4000 rpm for 10 min. Plasma was separated and stored at − 80 °C until further drug analysis.

#### Pharmacokinetic analysis

Pharmacokinetic parameters were evaluated using a non-compartmental model. The peak plasma concentration (Cmax) and the corresponding time (T_max_) were determined directly from the concentration–time curve. The area under the curve (AUC_₀–₄₈_ and AUC_₀–∞_) was calculated using the trapezoidal rule. The elimination rate constant (Kel) was derived from the slope of the log-linear phase of the plasma concentration–time curve, and the elimination half-life (T₁/₂) was calculated using Eq. 5, while the relative bioavailability was calculated using Eq. 6:

5 $$T\frac{1}{2}= \text{ } \mathrm{0.693/Kel}$$

6 $$\text{Relative bioavailability}=\frac{\text{AUC of formulation}}{\text{AUC of control}} \times 100$$

where AUC control is AUC_0-inf_ of either orally marketed BEN tablet, or BEN-DMNs.

### Statistical analysis

The required experimental data were analysed statistically with IBM SPSS Statistical software 21. One-way ANOVA was carried out with (95%) significance level, followed by Dunnett post-hoc test for the determination of the level of significance. The results significant if *p* < 0.05.

## Results and discussion

### Formulation and optimisation of BEN-TF

The transfersomal system was optimised through a Box–Behnken Design to study the impact of three formulation parameters: phospholipid concentration (Lipoid S75), surfactant level (sodium deoxycholate), and sonication time. These variables significantly influenced key responses which are vesicle size (Y₁), entrapment efficiency (Y₂), and transdermal flux (Y₃). The vesicle size ranged from 123.93 ± 1.06 nm to 167.1 ± 0.85 nm across the formulations, confirming nanoscale dimensions essential for transdermal penetration. EE% values varied between 95.0 ± 0.11% and 99.79 ± 0.03%, while transdermal flux spanned from 2.87 ± 0.22 to 9.85 ± 0.32 μg/cm^2^/hr. Following optimisation trials, the formulations' obtained dependent variables were fitted into the Box-Behnken Design's multiple models, with the quadratic model exhibiting the best fit and a coefficient of correlation (R^2^) almost equal to 1 Table 2.

**Table 2:** Actual and predicted value of dependent variables employed with BBD for BEN-TF formulations. (mean ± SD), n = 3

Formulation code	Independent variables			Dependent variables					
	A (mg)	B (mg)	C (sec)	Y1: vesicle size (nm)		Y2: EE (%)		Y3: Flux (μg/cm^2^/hr)	
				Actual	Predicted	Actual	Predicted	Actual	Predicted
BEN-TF1	300	30	35	146.8 ± 2.47	145.68	98.18 ± 0.22	97.41	9.61 ± 0.31	9.62
BEN-TF2	300	35	25	155.1 ± 0.85	155.29	97.82 ± 0.46	97.52	9.79 ± 0.37	9.69
BEN-TF3	300	30	35	146.2 ± 2.71	145.68	96.69 ± 0.21	97.41	9.55 ± 0.06	9.62
BEN-TF4	300	35	45	132.6 ± 0.35	133.67	95.3 ± 0.75	95.38	8.82 ± 0.14	8.65
BEN-TF5	350	35	35	145.5 ± 0.52	145.26	99.55 ± 0.13	99.58	2.87 ± 0.22	2.98
BEN-TF6	250	30	45	123.93 ± 1.06	123.89	96.87 ± 0.28	96.60	8.34 ± 0.15	8.34
BEN-TF7	250	30	25	151.23 ± 1.7	152.06	97.84 ± 0.16	97.95	8.63 ± 0.18	8.57
BEN-TF8	250	25	35	135.13 ± 3.75	135.37	99.29 ± 0.15	99.26	7.54 ± 0.25	7.43
BEN-TF9	300	25	45	128.7 ± 1.93	128.51	97.97 ± 0.27	98.27	9.66 ± 0.29	9.77
BEN-TF10	300	30	35	145.8 ± 1.25	145.68	96.65 ± 0.12	97.41	9.58 ± 0.59	9.62
BEN-TF11	300	30	35	144.1 ± 3.52	145.68	97.99 ± 0.32	97.41	9.52 ± 0.34	9.62
BEN-TF12	300	25	25	167.1 ± 0.85	166.03	98.58 ± 0.49	98.51	8.71 ± 0.28	8.88
BEN-TF13	300	30	35	145.5 ± 0.78	145.68	97.56 ± 0.5	97.41	9.85 ± 0.32	9.62
BEN-TF14	350	30	45	136.0 ± 0.79	135.17	99.12 ± 0.24	99.01	4.13 ± 0.31	4.19
BEN-TF15	250	35	35	127.66 ± 1.29	126.65	95.0 ± 0.11	95.19	7.01 ± 0.23	7.18
BEN-TF16	350	30	25	166.1 ± 1.14	166.15	99.79 ± 0.03	100.06	4.13 ± 0.17	4.13
BEN-TF17	350	25	35	141.1 ± 1.04	142.12	99.58 ± 0.19	99.39	3.21 ± 0.13	3.04

#### Response 1 (Y1): effect of independent variables on vesicles size of BEN-TF

The influence of the selected independent variables on vesicle size (Y1) was assessed using a fitted polynomial equation (Eq. 7), derived through the BBD:

7 $$\begin{array}{c}\mathrm{Y}1=145.68+6.34167\text{ A}-1.39575\mathrm{B}-14.7875\mathrm{C}+2.9665\mathrm{AB}-\\ 0.7\mathrm{AC}+3.975\mathrm{BC}-4.94417{\mathrm{A}}^{2}-3.38583{\mathrm{B}}^{2}+3.58083{\mathrm{C}}^{2}\end{array}$$

In this equation, A, B, and C correspond to the coded levels of lipoid S75 (mg), sodium deoxycholate (SDC) (mg), and sonication duration (sec), respectively. Based on regression analysis, a positive coefficient indicates a direct correlation with an enhancing or synergistic effect on the response, while a negative coefficient implies an inverse relationship or antagonistic effect [39]. Accordingly, the positive coefficient for lipoid S75 (A) signifies that increasing its concentration leads to an enlargement in vesicle size. In contrast, the negative coefficients for SDC (B) and sonication time (C) suggest that raising these factors results in a reduction in vesicle size.

To further validate the model, analysis of variance (ANOVA) was performed. The statistical significance of the model was confirmed by a high F-value and a p-value below 0.05, indicating that the model was a good fit [40]. These results are advantageous as they confirm a model to be a good fit for the corresponding response [41]. Additionally, the lack of fit was found to be non-significant, reinforcing that the variation is well explained by the model [42]. Among the tested variables and their interactions, A, B, C, AB, BC, A2, B2, and C2 were found to be statistically significant, whereas the interaction between A and C was not (p > 0.05). These findings indicate that all three independent factors significantly affect vesicle size, with lipoid S75 and sonication time showing a more pronounced effect (*p* < 0.0001), while SDC exhibited a comparatively smaller influence (*p* = 0.0131).

**Fig. 1:**
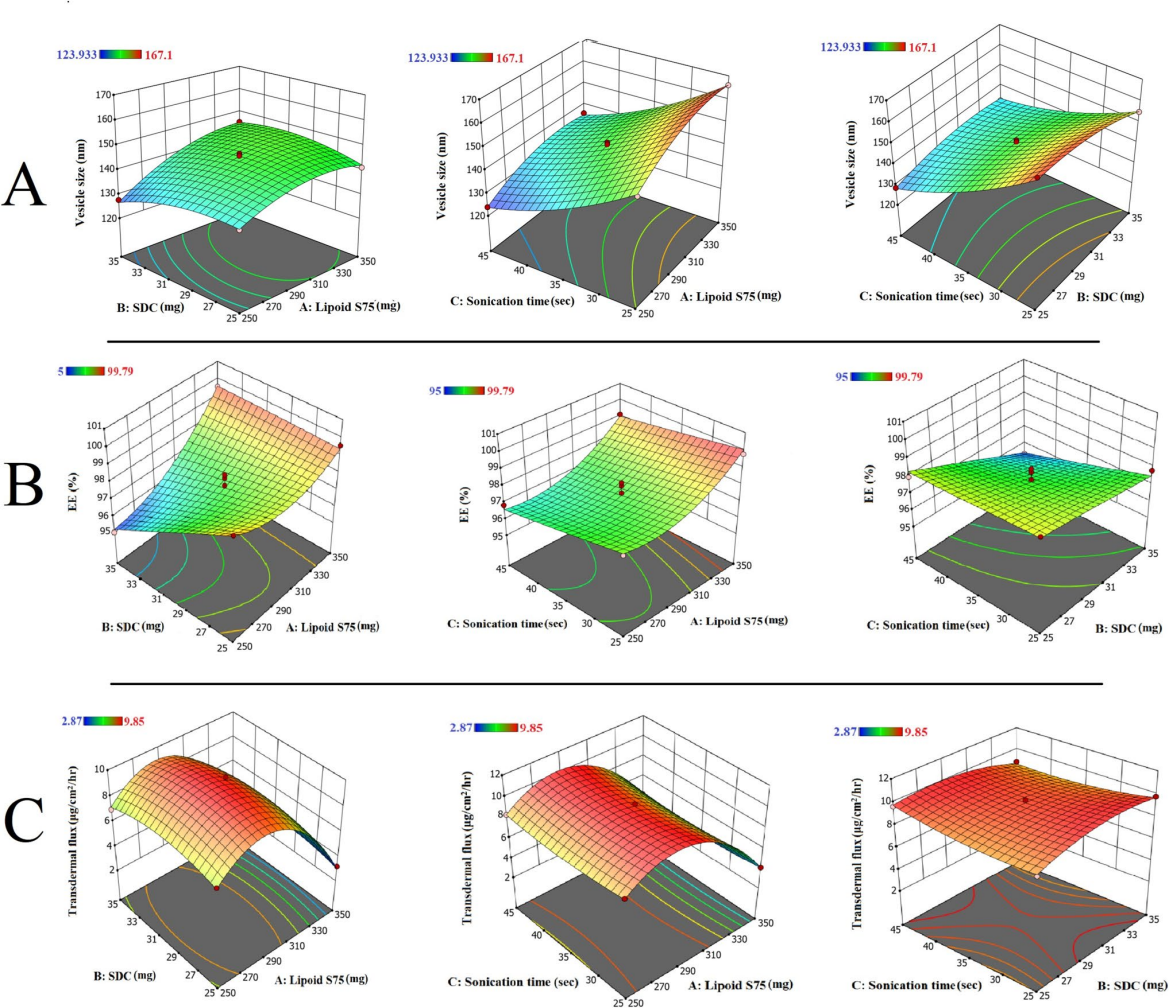
Three-dimensional response surface plots for representing comparative effects of independent variables on **A**) vesicle size, **B**) entrapment efficiency (EE%), and **C**) transdermal flux of BEN-TF

Figure 1(A) presents 3D response surface plots demonstrating how these variables affect vesicle size, which ranged between 123.93 ± 1.06 and 167.1 ± 0.85 nm for the BEN-TF formulations. Notably, all sizes were below 200 nm, aligning with the optimal range for transdermal drug delivery [43].The results show a clear trend of increasing vesicle size with elevated concentrations of lipoid S75. This observation is consistent with previous findings [44, 45], which can be attributed to the formation of multilamellar structures at higher lipid levels, leading to larger vesicles [46]. In contrast, increasing the amount of SDC and extending the sonication period led to smaller vesicles. The presence of charged molecules like SDC in the bilayer induces steric repulsion on the vesicle’s outer surface, thereby increasing membrane curvature and resulting in smaller vesicle sizes [47]. Furthermore, prolonged sonication helps in breaking down vesicle aggregates, further reducing size. For instance, BEN-TF6 and BEN-TF7, despite having identical compositions, exhibited different vesicle sizes of 123.93 ± 1.06 and 151.23 ± 1.7 nm, respectively. The observed difference was due to the longer sonication time (45 s) used in BEN-TF6 compared to BEN-TF7 (25 s), which contributed to the smaller vesicle size. Moreover, the contour plot and 3D illustrated a combination of two variables and assisted in identifying the optimal level of each variable as well as comprehending how the three variables interacted [48]. It is apparent that a lower concentration of lipoid S75 with a higher concentration of SDC, combined with longer sonication time, could produce the minimum vesicle size, such as in the case of BEN-TF6 and BEN-TF15 transfersome formulations.

Additionally, the PDI and zeta potential (ZP) were measured for BEN-TF formulations. PDI is a parameter used to represent size distribution, with a value of 0 denoting a fully homogeneous system and a value of 1 denoting a highly heterogeneous vesicle distribution [49]. Zeta potential, on the other hand, is a key indicator of colloidal stability, as it measures the degree of electrostatic repulsion between similarly charged particles in suspension [50]. A high magnitude of zeta potential whether strongly positive or negative suggests strong repulsive forces between particles, reducing the risk of aggregation. In contrast, low absolute ZP values imply weaker repulsive interactions, which may lead to particle aggregation or flocculation [51]. ZP values exceeding + 30 mV or below −30 mV are generally accepted as indicative of stable dispersions [52]. All BEN-TF formulations exhibited PDI values under 0.3, denoting a uniform size distribution that is suitable for phospholipid-based carriers [53]. Additionally, the zeta potential measurements revealed that the transfersomes carried a strong negative surface charge, ranging from −30.36 ± 2.30 mV to −43.91 ± 1.80 mV. These values confirm the colloidal stability of the formulations. The pronounced negative charge is attributed to the inclusion of sodium deoxycholate (SDC), an anionic surfactant that contributes to the overall surface charge of the vesicles [54].

#### Response 2 (Y2): effect of independent variables on entrapment efficiency (EE%) of BEN-TF

The effect of independent variables on the entrapment efficiency (EE%) of BEN-TF formulations was expressed by a fitted polynomial model (Eq. 8):

8 $$\begin{array}{c}\mathrm{Y}2=97.414+1.13\mathrm{A}-0.96875\mathrm{B}-0.59625\mathrm{C}+1.065\mathrm{AB}+\\ 0.075\mathrm{AC}-0.4775\mathrm{BC}+0.96425{\mathrm{A}}^{2}-0.02325{\mathrm{B}}^{2}+0.02675{\mathrm{C}}^{2}\end{array}$$

In this equation, the coded factors A, B, and C correspond to the amount of lipoid S75 (mg), sodium deoxycholate (SDC) (mg), and sonication time (sec), respectively. As indicated by the equation, a higher concentration of lipoid S75 (A) positively influences EE%, suggesting improved drug entrapment with increasing lipid content. Conversely, increasing either SDC (B) or sonication time (C) negatively affects EE%, resulting in reduced drug entrapment. Statistical analysis confirmed that the model is significant, with a non-significant lack of fit, indicating a good fit between predicted and observed values. Among the terms assessed, the individual factors A, B, and C, the interaction term AB, and the quadratic term A^2^ showed a statistically significant influence on EE% (*p* < 0.05), while the remaining terms were found to be non-significant.

The response surface plots in Fig. 1(B) depict the influence of these variables on EE%. Entrapment efficiency is a key parameter for evaluating formulation performance, as it reflects the capacity of the system to encapsulate the active compound [55]. Among the tested formulations, BEN-TF16 exhibited the highest EE% (99.79 ± 0.03%), while BEN-TF15 demonstrated the lowest (95.0 ± 0.11%). A notable increase in EE% was observed when the lipoid S75 concentration rose from 250 to 300 mg. This can be attributed to the enhanced solubilization and encapsulation of benidipine, a highly lipophilic drug, within the lipid matrix [44, 56]. Furthermore, higher lipid content can lead to the formation of multilamellar vesicles, which are known to accommodate larger amounts of drug molecules [57, 58].

In contrast, elevating the amount of SDC from 25 to 35 mg led to a reduction in EE%. This decline could be linked to the formation of mixed micelles coexisting with vesicles at higher surfactant concentrations, which possess smaller sizes and rigid structures, thereby limiting drug encapsulation [8, 59].When the surfactant concentration surpasses its critical micelle concentration (CMC), it tends to disrupt the phospholipid bilayer, leading to the formation of non-vesicular aggregates like micelles, which further diminishes drug loading capacity [60]. Hence, the type and concentration of surfactant are critical factors in achieving optimal vesicle characteristics [61]. This observation aligns with findings by Patel et al., who investigated curcumin-loaded transfersomes and reported that EE% decreased with increased surfactant levels but improved with higher phospholipid content [62].

In another study, phenylethyl resorcinol-loaded transfersomes formulated with SDC reached an EE% of approximately 95.20%. Given that SDC is an anionic surfactant, its interaction with positively charged drugs can enhance entrapment through electrostatic attraction, provided the concentrations are within optimal limits [63]. Many factors can affect entrapment efficiency. For instance, a higher lipid concentration was associated with a higher entrapment efficiency, and if the surfactant concentration rises above a specific point, the EE% falls [64]. Furthermore, it was found that there was a reverse relationship between the sonication time and entrapment efficiency, with the EE% decreasing as the sonication time increased and vice versa. This outcome concurs with previous research findings [65].

#### Response 3 (Y3): effect of independent variables on transdermal flux (Jss) of BEN-TF

The effect of formulation variables on the transdermal flux (Jss) of BEN-TF was analysed using the polynomial model (Eq. 9):

9 $$\begin{array}{c}\mathrm{Y}3=9.622-2.1475\mathrm{A}-0.07875\mathrm{B}-0.03875\mathrm{C}+0.0475\mathrm{AB}+\\ 0.0725\mathrm{AC}-0.48\mathrm{BC}-3.701{\mathrm{A}}^{2}-{0.7635\mathrm{B}}^{2}+{0.3865\mathrm{C}}^{2}\end{array}$$

In this equation, A, B, and C represent the coded values for the amount of lipoid S75 (mg), sodium deoxycholate (SDC) (mg), and sonication time (sec), respectively. All coefficients in Eq. 9 were negative, indicating that each independent variable exerted an antagonistic effect on transdermal flux. In other words, increasing the levels of lipoid S75, SDC, or sonication time individually led to a decrease in the drug permeation rate through the skin. However, based on ANOVA results, the amount of lipoid S75 (A) had a statistically significant effect on transdermal flux (*p* < 0.05), while the effects of SDC (B) and sonication time (C) were not statistically significant (*p* > 0.05). Among the interaction and quadratic terms, BC, A^2^, B^2^, and C^2^ were found to have a significant influence on Jss, whereas the other interaction terms did not show statistical significance. The model was deemed statistically valid, supported by a significant model F-value and a non-significant lack of fit, which confirms the model's adequacy in representing the experimental data.

Ex-vivo permeation studies provide crucial insight into the potential in vivo performance of transdermal formulations, as they reflect the amount of drug capable of being absorbed through the skin barrier [66]. The J_ss_ values observed across the 17 experimental runs ranged between 2.87 ± 0.22 and 9.85 ± 0.32 μg/cm^2^/hr. As shown in the 3D response surface plot (Fig. 1, C), transdermal flux increased with rising lipoid S75 concentrations from 250 to 300 mg. This outcome suggests that, although the polynomial model indicated a negative linear effect, interaction and quadratic effects likely contributed to the observed enhancement in flux at higher lipid concentrations. Nevertheless, a significant reduction in the transdermal flow occurs when the lipoid S75 concentration is raised from 300 to 350 mg. The impact of SDC concentration on transdermal flux was negligible (*p*-value < 0.05), although 30 mg of SDC provided a higher transdermal flux as well, and the sonication time between 25 and 45 s showed similar transdermal flux. The fact that the BEN-loaded transfersome sizes were less than 170 nm could contribute to clarifying this, as lipid vesicles with diameters ≤ 300 nm have been shown to be able to permeate deeper layers of the skin [67]. Furthermore, the addition of surfactant to the vesicle bilayer lipid membranes provided transfersomes exceptional flexibility, allowing them to squeeze through extremely tiny skin barrier constrictions [64, 68]. Surfactant is crucial for transfersomes that act as permeation enhancers by loosening or destabilising the membrane's lipid bilayer [69, 70]. In comparison to other surfactants such as sodium cholate and Briz-35, SDC demonstrated superior skin penetration. This could be because of its improved interaction with phospholipid bilayers, resulting in greater deformability and improved skin penetration [71].

#### Optimisation of BEN-TF based on the desirability criterion

Design-Expert^®^ software provides a platform for formulation optimisation using the desirability function approach. This method employs polynomial equations to quantitatively define the relationships between independent variables and corresponding responses [72]. By applying the desirability function, it is possible to concurrently determine the optimal levels of multiple input variables, thereby identifying conditions that yield the most favourable outcomes for one or more response parameters [73, 74]. To achieve the minimum vesicle size, maximum entrapment efficiency (EE%), and maximum transdermal flux, Design-Expert^®^ software recommended an optimised formulation with an overall desirability score of 0.893.

To validate the optimum conditions, triplicate experiments were conducted using the optimised formulation. This formulation consisted of lipoid S75 (263.56 mg), SDC (25 mg), and a sonication time of 45 s. The results obtained under these conditions closely matched the predicted values from the optimisation analysis performed using desirability functions, particularly for vesicle size, entrapment efficiency, and transdermal flux, as detailed in Table 3. These findings support the effectiveness of the BBD, combined with the desirability function approach, as a reliable strategy for optimising BEN-TF formulations. The polydispersity index (PDI) and zeta potential of the optimised BEN-TF were found to be 0.201 ± 0.011 and –31.60 ± 1.77 mV, respectively. Additionally, the BEN content in the optimised formulation was determined to be 100.43 ± 0.12%.

**Table 3:** Optimum formulation conditions, and comparison of predicted and experimental values of response variables for the optimised BEN-TF formulation

Optimum independent variable	Coded levels	Actual levels
A: Amount of lipoid S75 (mg)	˗ 0.73	263.56
B: Amount of SDC (mg)	˗ 1	25
C: Sonication time (sec)	+ 1	45
Dependent variables	Predicted values	Experimental values
Y1: Vesicles size (nm)	123.933	124.9 ± 1.49
Y2: Entrapment efficiency (%)	98.67	98.12 ± 0.18
Y3: Transdermal flux (μg/cm^2^/hr)	9.34	9.74 ± 0.53

The enhancement ratio (ER), which reflects the extent of improvement in transdermal flux of the test formulations compared with control formulations, was calculated using the transdermal flux of the optimised BEN-TF (9.74 ± 0.53 μg/cm^2^/hr). The control formulations included conventional liposomes (BEN-CL) and a hydroethanolic solution (BEN-HE). The transdermal flux values for BEN-CL and BEN-HE were 1.63 ± 0.25 and 1.13 ± 0.14 μg/cm^2^/hr, respectively. The optimised BEN-TF formulation demonstrated superior drug permeation performance, exhibiting enhancement ratios (ERs) of 5.97-fold and 8.61-fold compared with BEN-CL and BEN-HE, respectively. The flexibility differential between the transfersome formulation and conventional liposome might be responsible for the transfersome formulation's superior performance. When the transfersome vesicles penetrate the SC layer of skin, they can squeeze through pores that are far smaller than their diameters [43]. Transfersomal vesicles would be able to pass through the membranes' tiny holes because the surfactant would reduce the energy required for deformation [75].

#### Differential scanning calorimetry (DSC)

**Fig. 2:**
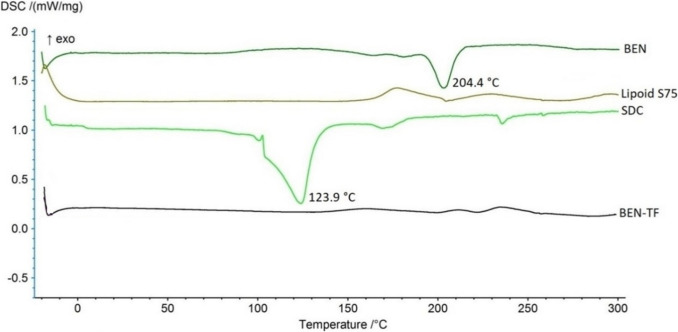
DSC thermograms of pure BEN, lipoid S75, SDC and the optimised BEN-TF formulation

Differential Scanning Calorimetry (DSC) studies were performed to assess the crystallinity of BEN within the formulations and to evaluate any potential interactions between BEN and the excipients. The DSC thermograms of pure BEN, lipoid S75, sodium deoxycholate (SDC), and the optimised BEN-TF formulation are shown in Fig. 2. Pure BEN exhibited a sharp melting endotherm at 204.4 °C, which is consistent with previously reported melting points ranging from 203 °C to 204.9 °C [76, 77]. Lipoid S75 did not exhibit any distinct endothermic melting temperature within the studied temperature range, which aligns with earlier findings [78, 79]. However, the exothermic signal observed at higher temperatures could be attributed to the onset of thermo-oxidative/decomposition processes characteristic of unsaturated soy phosphatidylcholine mixture [80, 81]. Conversely, SDC exhibited a broad endothermic event initiating at approximately 110.82 °C, likely due to the loss of water molecules, followed by a distinct sharp endothermic peak at 123.9 °C [82, 83]. This behaviour is consistent with previous TGA studies, which confirmed that the first thermal event corresponds to the loss of four crystallisation water molecules. Supporting evidence from X-ray diffraction and density measurements showed that residues after this step appeared amorphous but regained their crystalline structure upon rehydration. Furthermore, comparable DSC thermal behaviour has been reported for DSC crystals and fibres [84]. Notably, the DSC thermogram of the optimised BEN-TF formulation showed the complete disappearance of BEN’s characteristic melting peak, indicating a loss of its crystalline structure. This observation suggests that BEN was converted into an amorphous form within the lipid vesicle matrix.

#### Attenuated total reflectance spectroscopy (ATR- FTIR)

**Fig. 3:**
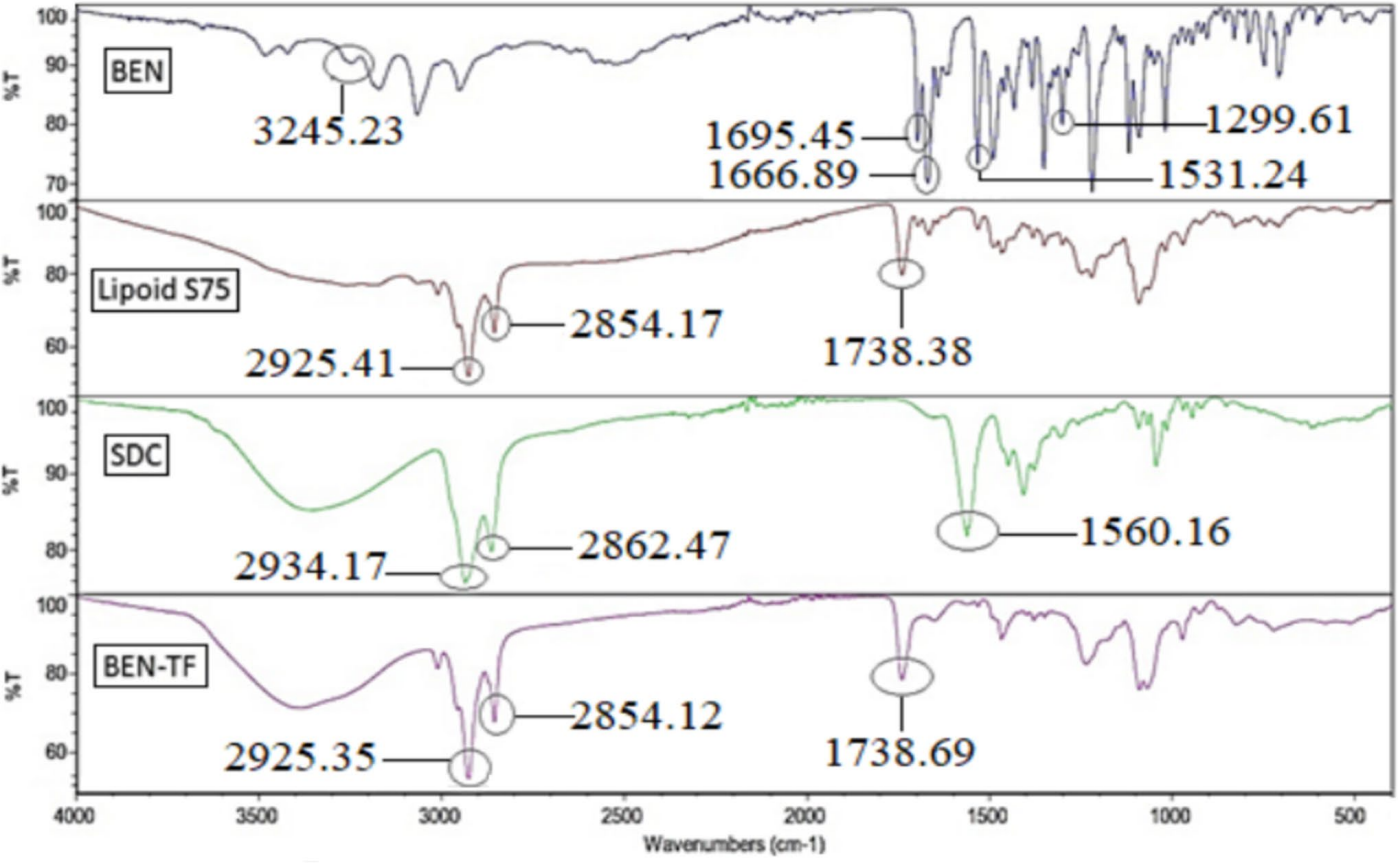
ATR-FTIR spectrum of pure BEN, lipoid S75, and the optimised BEN-TF formulation

ATR-FTIR spectroscopy was employed to investigate potential interactions between BEN and the excipients. The FTIR spectra of pure BEN, lipoid S75, SDC, and the optimised BEN-TF formulation are presented in Fig. 3. The spectrum of pure BEN exhibited a distinct absorption band at 3245.23 cm⁻^1^, attributed to N–H stretching vibrations of the dihydropyridine ring. Two prominent absorption bands at 1695.45 cm⁻^1^ and 1666.89 cm⁻^1^ were likely due to the stretching vibrations of the carbonyl (C = O) groups within the carboxyl moieties of the side chain. Additionally, a strong band at 1531.24 cm⁻^1^ was associated with NO₂ stretching, indicative of the nitrophenyl group, while the sharp absorption band at 1299.61 cm⁻^1^ corresponded to the C–N stretching vibration. These characteristic peaks were in agreement with previously reported spectra of BEN [76, 85].

The FTIR spectrum of lipoid S75 showed intense absorption peaks at 2925.41 cm⁻^1^ and 2854.17 cm⁻^1^, corresponding to the antisymmetric and symmetric stretching of CH₂ acyl chains, respectively. A strong absorption at 1738.38 cm⁻^1^ was attributed to ester carbonyl (C = O) stretching [78]. In the case of SDC, peaks were observed at 2934.17 cm⁻^1^, 2862.47 cm⁻^1^, and 1560.16 cm⁻^1^, which represent the stretching vibrations of CH and COO⁻ groups [86, 87]. The FTIR spectrum of the optimised BEN-TF formulation showed significant changes compared with that of the pure drug, including shifts, overlaps, and the disappearance of certain characteristic BEN peaks. The characteristic BEN band at 3245 cm⁻^1^ (N–H stretching) overlaps with the broad absorption region around 3500 cm⁻^1^ in both Lipoid S75 and SDC. This broad feature corresponds to hydrogen-bonded O–H stretching [88, 89]. The BEN carbonyl bands at 1695.45 and 1666.89 cm⁻^1^ are likewise overlapped and become less distinct in the BEN-TF spectrum, which might be attributed to hydrogen bonding between phospholipid headgroups and the BEN carbonyls. A similar effect has been reported for indomethacin, whose characteristic bands at 1720 and 1690 cm⁻^1^ disappeared due to hydrogen bonding with the phospholipid carbonyl group [90]. The NO₂ stretching and C–N stretching vibration (1531 and 1299 cm⁻^1^) also diminished or disappeared, suggesting that the drug is molecularly incorporated into the transfersome. The absence of some BEN-specific bands in the formulation spectrum, along with the persistence of key lipoid S75 peaks at 2925 cm⁻^1^, 2854 cm⁻^1^, and 1738 cm⁻^1^, supports the successful entrapment of BEN within the lipid vesicles and its association with the lipid matrix. Consistently, Malviya et al. observed retention of major phospholipid peaks upon drug loading in transferosomes/ethosomes and concluded that the disappearance of drug-specific bands evidences encapsulation [91].

#### X-ray diffraction (XRD)

**Fig. 4:**
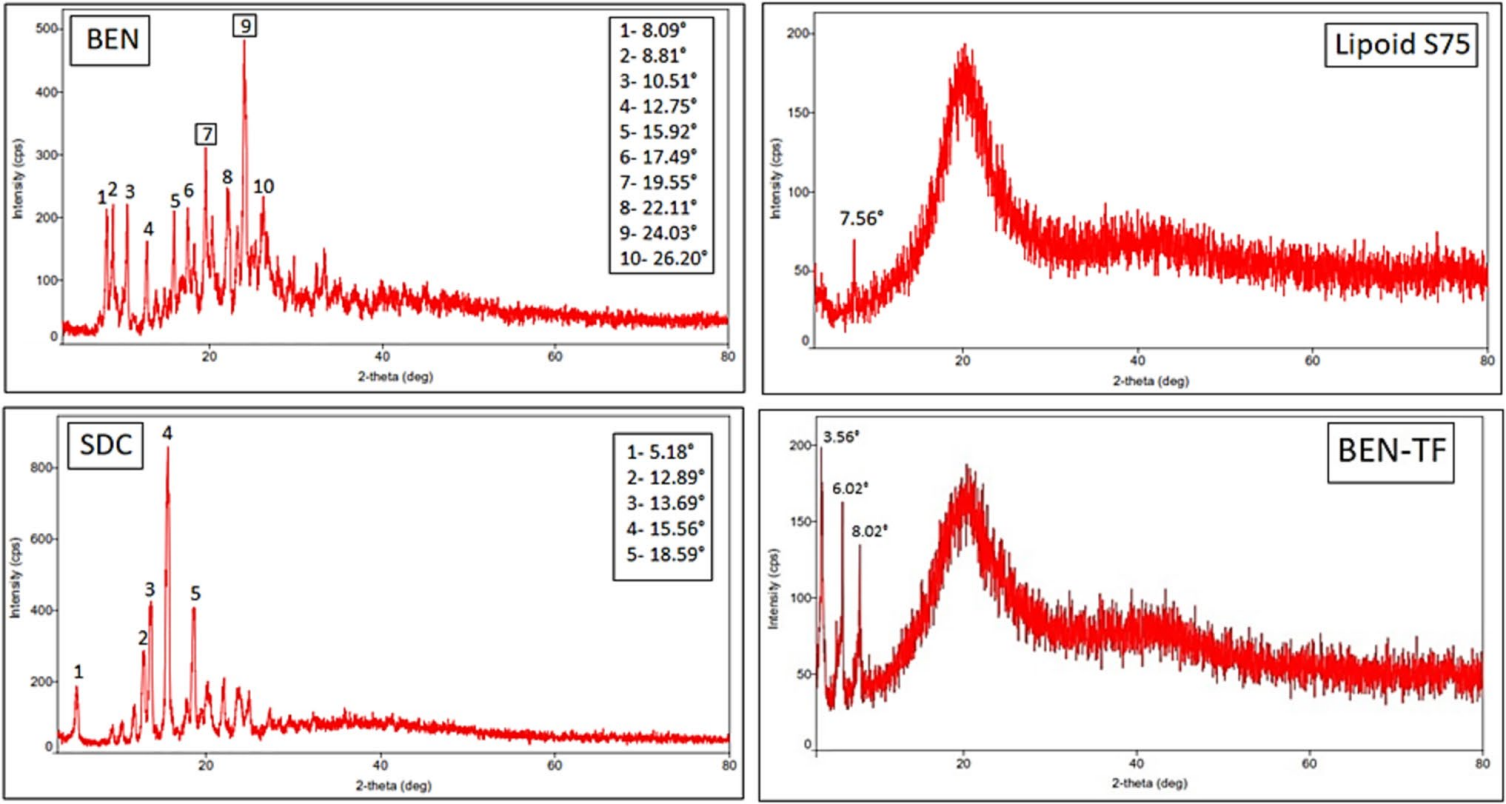
XRD patterns of pure BEN, lipoid S75, sodium deoxycholate (SDC), and optimised BEN-TF

To investigate potential transformations and changes in crystallinity in BEN-TF formulation, XRD analysis was carried out on pure BEN, lipoid S75, and the optimised BEN-TF formulation, as illustrated in Fig. 4. The diffractogram of pure BEN exhibited sharp, narrow, and intense peaks reflecting its highly crystalline nature. This finding is consistent with previously reported diffraction patterns of BEN [85]. Lipoid S75 showed a single intense peak that also aligns with earlier reports [78, 92]. In the case of the optimised BEN-TF formulation, the characteristic crystalline peaks of BEN were either significantly diminished or completely suppressed, with only a few weak peaks observed between 3° and 8° at 2θ angles. This reduction in peak intensity and number suggests that BEN underwent a phase transformation from a crystalline to an amorphous state upon incorporation into the transfersomal system.

#### Field emission scanning electron microscopy (FESEM) and high-resolution transmission electron microscopy (HRTEM)

**Fig. 5:**
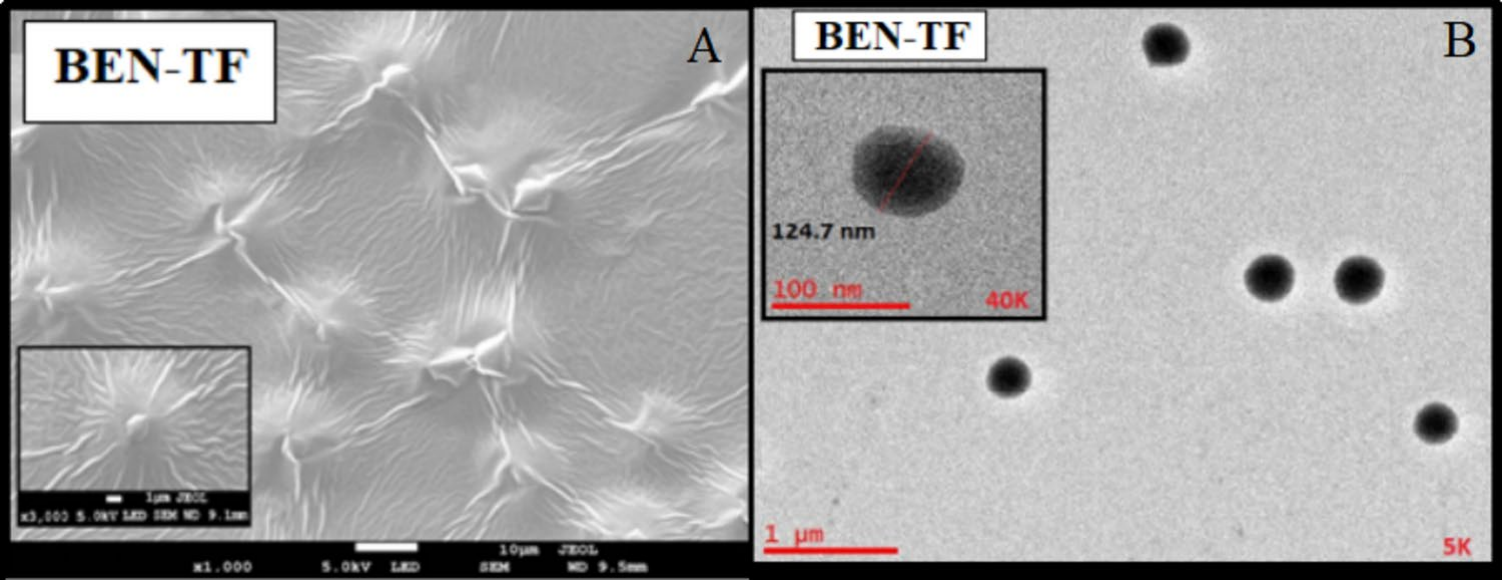
**A**) FESEM image of the optimised BEN-TF formulation, and **B**) HRTEM Images of the optimised BEN-TF formulation with magnifications power of 30kx and 60kx operating at 200kv

The surface morphology of the optimised BEN-TF formulation was examined using FESEM, as shown in Fig. 5(A). The vesicles appeared largely uniform, with a predominance of spherical shapes and an overall acceptable distribution of lipid vesicles. The HRTEM images of the same formulation, presented in Fig. 5(B), further confirmed the spherical morphology, with no visible signs of vesicle rupture or structural damage. The vesicles retained their integrity and displayed a consistent shape. The particle size of the optimised BEN-TF was approximately 124.7 nm. Such nanoscale vesicular systems are considered advantageous for drug delivery, as they can enhance drug bioavailability, modify biodistribution, and allow for controlled release [93]. In particular, nanosized lipid vesicles are well suited for transdermal drug delivery, as they facilitate deeper skin penetration [94]. It is generally accepted that an optimal vesicle size for effective transdermal delivery is below 200 nm [95].

#### Confocal laser scanning microscopy (CLSM)

**Fig. 6:**
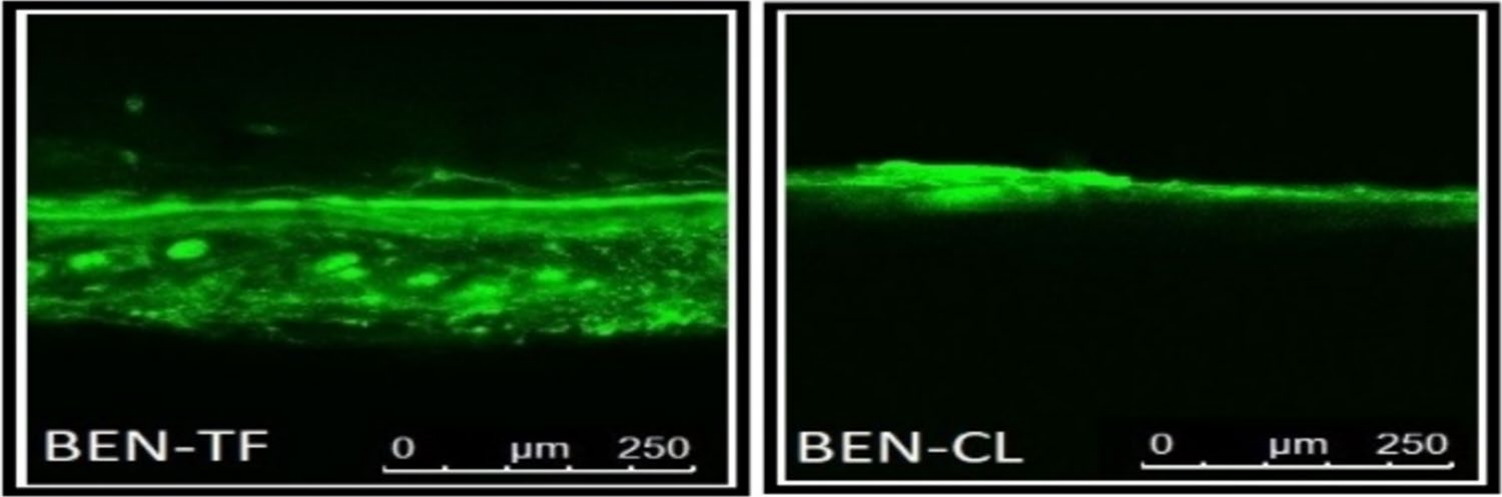
CLSM images of optimised BEN-TF and BEN-CL formulation

The CLSM study was conducted to assess the extent of skin penetration achieved by the optimised BEN-TF formulation in delivering BEN to deeper skin layers. Fluorescent images of the optimised BEN-TF and the conventional liposome formulation (BEN-CL) are presented in Fig. 6. The BEN-TF images demonstrated stronger and more uniform fluorescence across the stratum corneum (SC), viable epidermis, and dermis, indicating enhanced penetration compared with the BEN-CL formulation. In contrast, BEN-CL showed limited penetration, with fluorescence primarily localised to the upper skin layers and forming a superficial reservoir, both in terms of intensity and depth. To quantify dye penetration depth and intensity, the CLSM images were further analysed using ImageJ software, and the results are summarised in Table 4. The observed difference in penetration can be attributed to the structural properties of the formulations. Liposomes possess a relatively rigid bilayer structure, whereas transfersomes being composed of phospholipids and edge activators exhibit enhanced elasticity and deformability. This flexibility allows transfersomes to better navigate through the intercellular pathways of the skin, thereby facilitating deeper drug delivery [96].

**Table 4:** Confocal microscopy shows the value of intensity and depth of formulations across the rat skin. (mean ± SD), *n* = 3

Treated formulation with the rat skin	Intensity (gray value)	Penetration Depth (µm)
BEN-TF	100.71 ± 10.02	186.42 ± 8.05
BEN-CL	37.11 ± 4.39	80.82 ± 6.56

### Optimised BEN-TF formulation integrated into dissolving microneedles (BEN-TF-DMNs)

The optimised BEN-TF formulation was integrated into dissolving microneedles (DMNs) prepared using hyaluronic acid (HA) to enhance the formulation's stability, permeability, and handling without postponing BEN release [97].

#### Morphology study

**Fig. 7:**
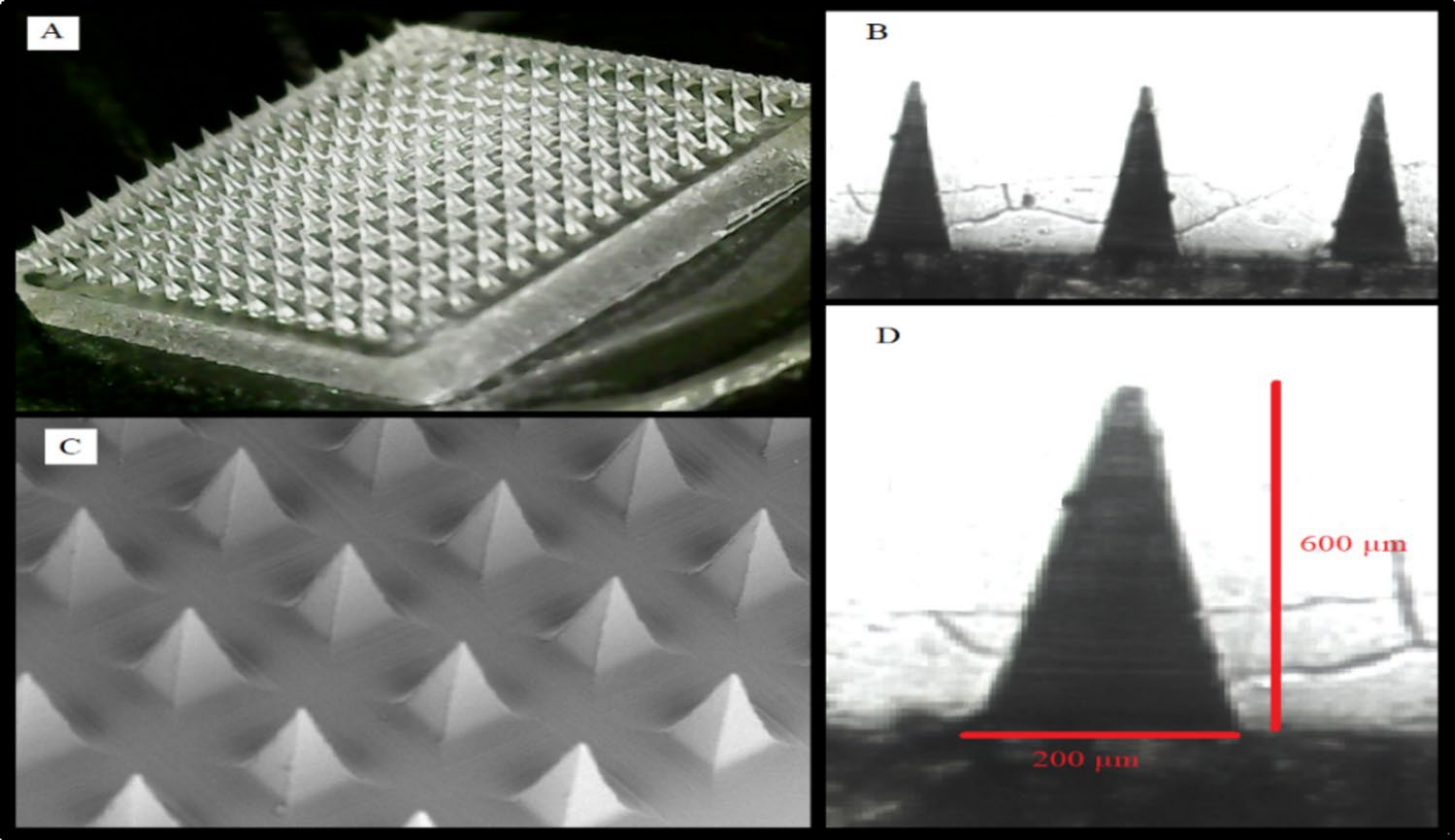
Morphology of BEN-TF-DMNs (**A**) digital microscope, (**B**) inverted microscope (4X), (**C**) SEM, and (**D**) inverted microscope (20X) with measurements

The morphological characteristics of the optimised BEN-TF-loaded dissolving microneedles (DMNs) are shown in Fig. 7. Inverted microscopy revealed well-defined, pyramidal micro-projections, with each needle measuring approximately 600 µm in length and 200 µm in base width, arranged in a 15 × 15 array. Additionally, Scanning Electron Microscopy (SEM) confirmed the presence of uniformly shaped, sharp pyramidal microneedles, supporting the structural integrity and precision of the fabricated DMNs.

#### Mechanical Strength of BEN-TF-DMNs

**Fig. 8:**
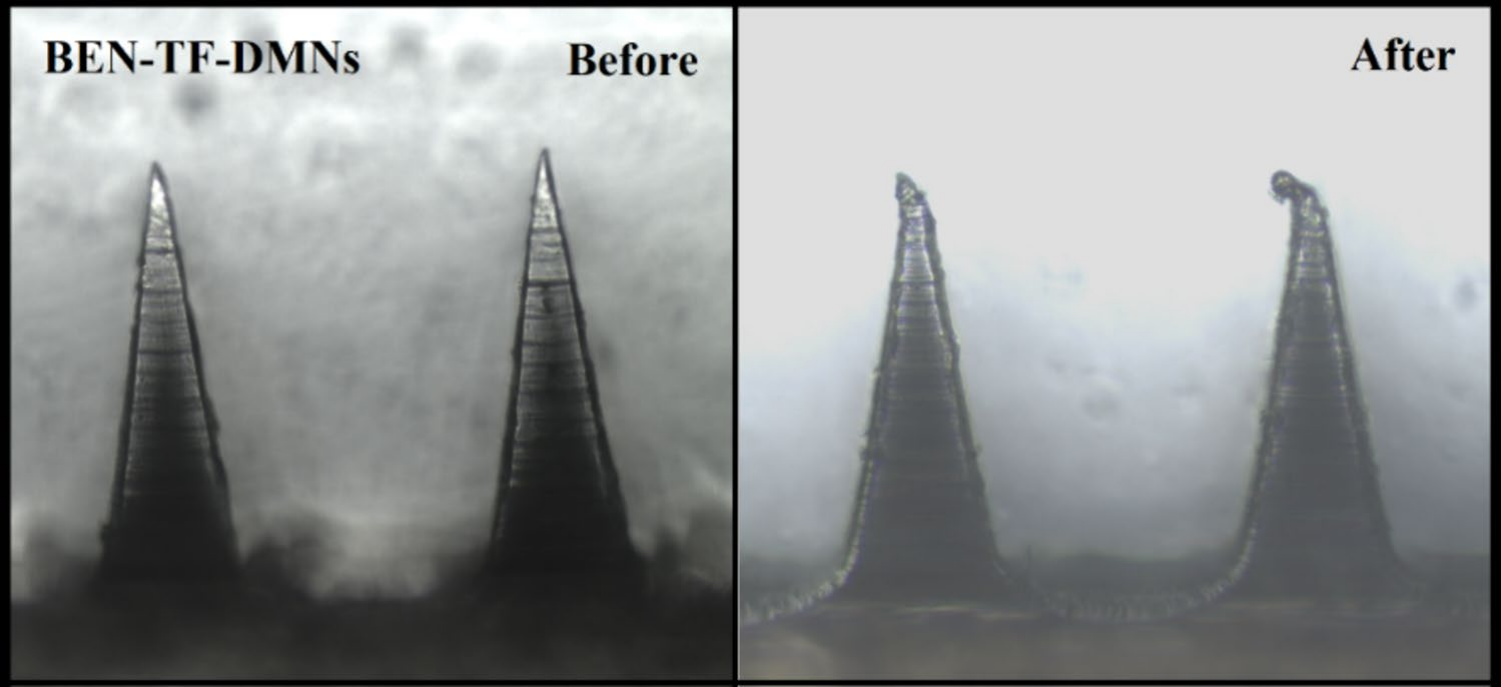
The images of optimised lipid vesicles-loaded DMNs before and after mechanical test

The mechanical strength of the BEN-TF-DMNs was assessed using a texture analyser. Upon compression, the optimised DMNs exhibited a height reduction of 5.62 ± 2.56% relative to their original length. As shown in Fig. 8, the microneedle tips became slightly blunted after compression but did not display any visible fractures. These results indicate that the DMN arrays possess sufficient mechanical strength to resist deformation under applied force. It is important to note that the compression tests were conducted against a rigid metal surface, which is significantly less yielding than biological tissue. Therefore, the microneedles are expected to perform even more effectively when applied to skin, which is considerably softer and more elastic [98].

#### Insertion capability of BEN-TF-DMNs

**Fig. 9:**
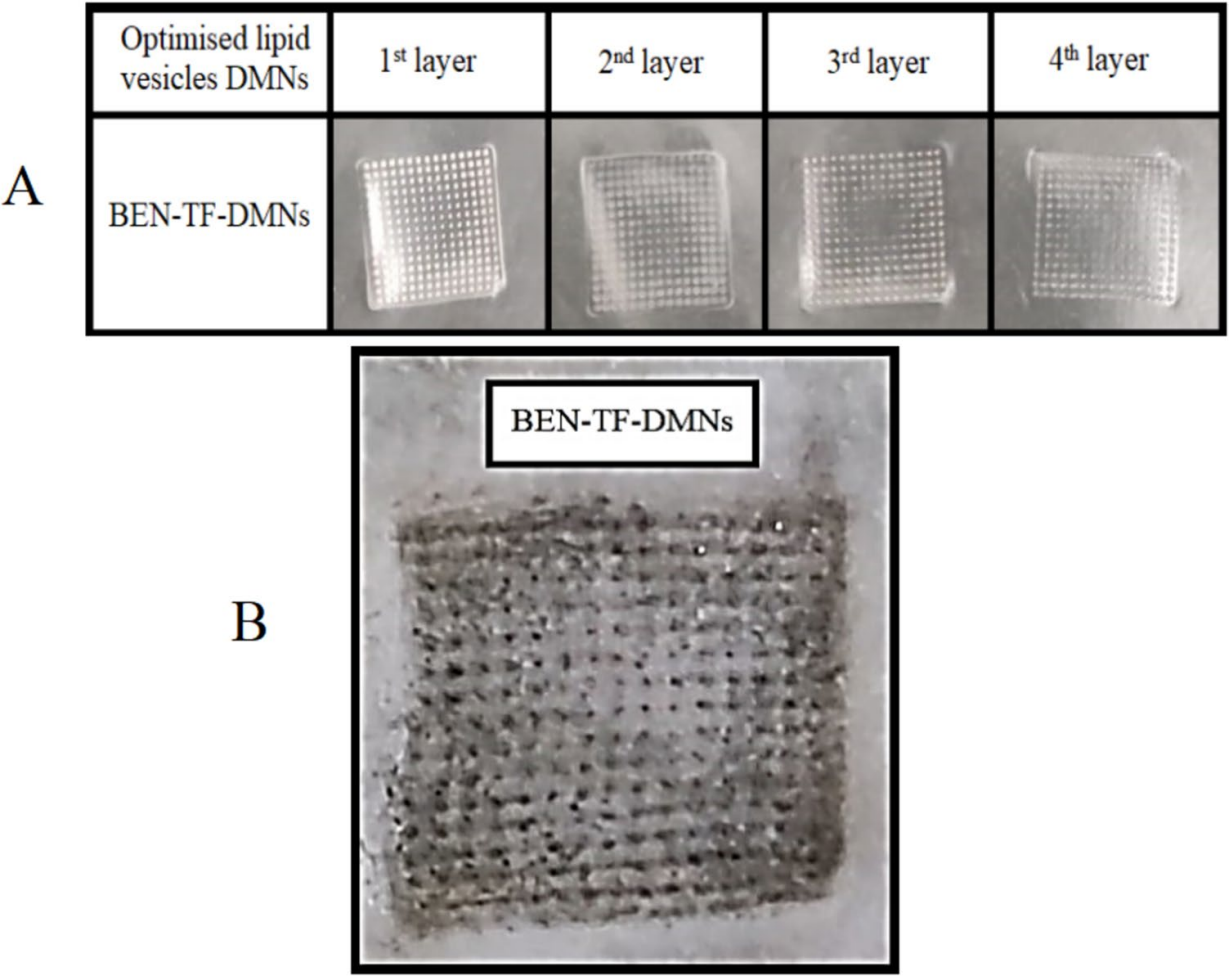
**A**) Holes created in each Parafilm M^®^ layer for BEN-TF-DMNs, **B**) View of the skin's surface after insertion of BEN-TF-DMNs

The insertion ability of the optimised BEN-TF-DMN arrays was evaluated using Parafilm^®^ M as a skin simulant. Eight layers of Parafilm^®^ were stacked, and the DMNs were inserted manually. Following insertion, each Parafilm^®^ layer was separated and visually inspected to assess the formation and consistency of microchannels as an indicator of penetration depth. The BEN-TF-DMNs successfully penetrated up to four layers of Parafilm^®^, as demonstrated in Fig. 9(A). The number of visible microchannels decreased progressively with increasing depth. Further quantitative analysis, shown in Fig. 9(B), revealed that approximately 100% of the microneedles pierced the first and second layers, while the percentage decreased in the third and fourth layers, indicating a gradual reduction in penetration depth. The insertion capability was also assessed in excised rat abdominal skin. As shown in Fig. 9 (B), the microneedles created distinct micropores upon insertion, confirming their ability to breach the stratum corneum (SC) effectively. These results suggest that the BEN-TF-loaded DMNs possess sufficient mechanical strength and structural integrity to penetrate the SC and potentially deliver the encapsulated drug to deeper skin layers.

#### Dissolution of BEN-TF-DMNs

**Fig. 10:**
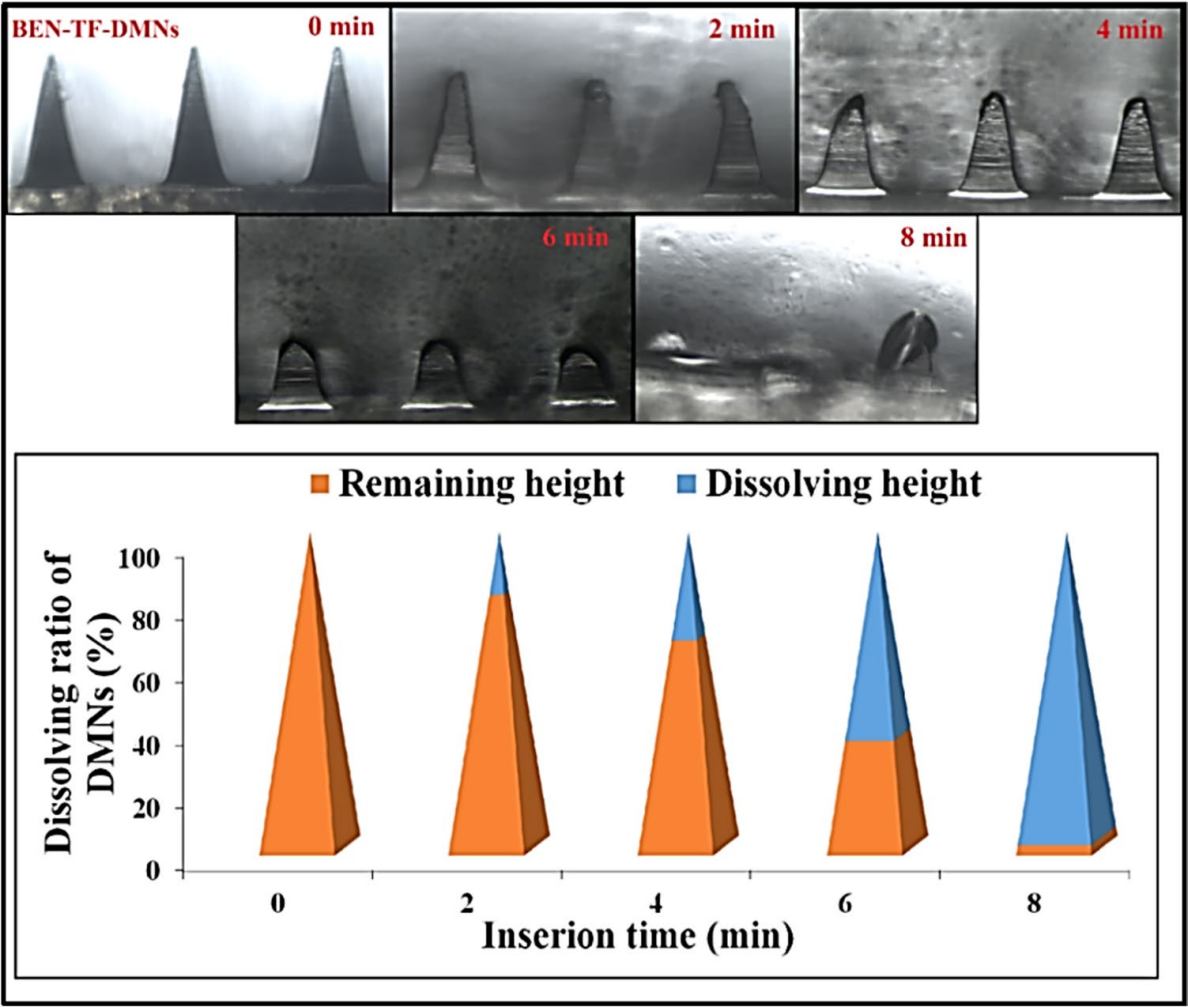
Images of dissolution of BEN-TF-DMNs after insertion into rat skin, and their dissolving ratio along with insertion time. (mean ± SD), *n* = 3

The dissolution behaviour of the BEN-TF-DMNs was assessed at time intervals of 2, 4, 6, and 8 min, as illustrated in Fig. 10. The DMNs exhibited a progressive dissolution profile over time. The percentage of the formulation dissolved was approximately 19.52 ± 1.25%, 33.50 ± 2.43%, 64.52 ± 2.25%, and 96.79 ± 1.65% at 2, 4, 6, and 8 min, respectively. These findings indicate that the DMNs gradually dissolved upon application and were almost entirely dissolved within 8 min of insertion into rat skin. This confirms their self-dissolving nature, suggesting that the system is capable of rapidly releasing the encapsulated drug upon administration to the skin.

#### BEN content in BEN-DMNs and BEN-TF-DMNs

The drug content analysis revealed that the BEN-DMNs contained 151.49 ± 5.17 µg of BEN, whereas the BEN-TF-DMNs incorporated a lower amount of 59.12 ± 6.52 µg. The reduced drug loading observed in the BEN-TF-DMNs may be attributed to the increased viscosity of the final polymer blend resulting from the inclusion of lipid vesicles. This increased viscosity could hinder the complete filling of the micromould cavities, thereby affecting the overall drug loading efficiency. This phenomenon has been previously reported in the literature and is commonly associated with DMNs loaded with lipid-based vesicular systems [99, 100]. Demartis et al. [99] reported that while 139.31 ± 22.16 µg of rose bengal were loaded in conventional DMNs, only 64.37 ± 8.61 µg were successfully loaded when using transfersome-integrated DMNs.

#### Ex-vivo skin permeation study of BEN-TF-DMNs

**Fig. 11:**
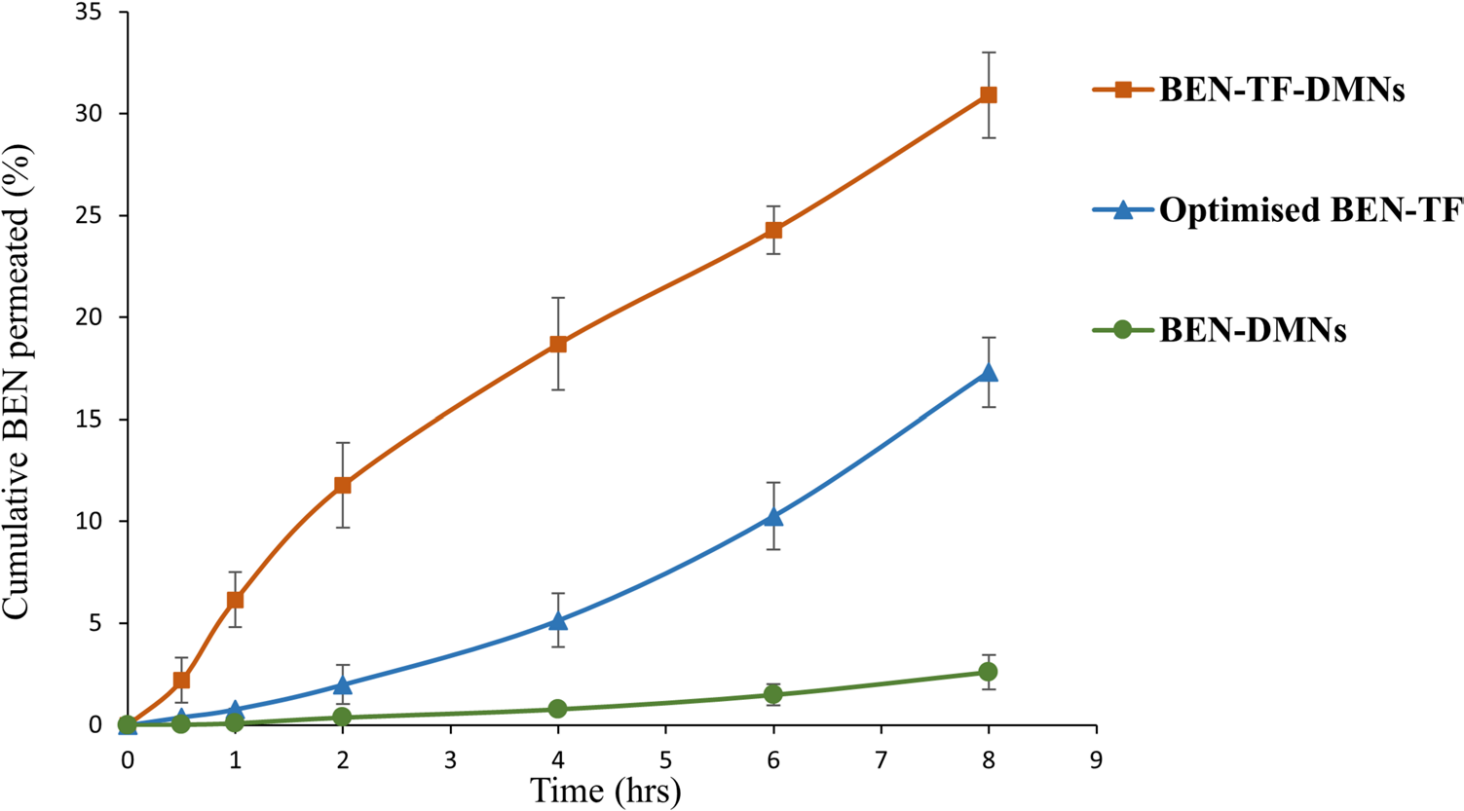
Permeation profiles of BEN-DMNs, BEN-E, and BEN-TF-DMNs

The permeation profiles of the optimised BEN-TF, BEN-DMNs, and BEN-TF-DMNs were evaluated using excised rat skin over 8 h, as presented in Fig. 11. The transdermal flux of the BEN-TF-DMNs was found to be 5.23 ± 0.64 µg/cm^2^/hr, significantly higher than that of the BEN-DMNs, which was 1.64 ± 0.32 µg/cm^2^/hr. These results indicate that the design and optimisation of an appropriate carrier system can substantially enhance the skin permeation of BEN. Transfersomes' composition enhances flexibility and deformability, which enables them to traverse the skin’s narrow intercellular spaces [101]. In addition, the integration of transfersomes with dissolving microneedles (DMNs), which create microchannels in the stratum corneum, further increases the drug permeability through the skin. As a result, the BEN-TF-DMNs showed a significantly improved permeation profile compared to BEN-DMNs alone, with an enhancement ratio of 3.19 ± 0.20.

Furthermore, the calculated permeation coefficients for the formulations were as follows: BEN-TF at 0.0081 ± 0.0021 cm/hr, BEN-DMNs at 0.0108 ± 0.0011 cm/hr, and BEN-TF-DMNs at 0.0884 ± 0.0162 cm/hr. Notably, the permeation coefficient of the BEN-TF-DMNs was substantially higher than those of the other two formulations. The permeation coefficient is a critical parameter widely used in comparative studies of transdermal delivery systems, reflecting the ability of a formulation to distribute the drug into the deeper layers of the skin [102–104].These findings highlight the synergistic potential of combining ultradeformable lipid vesicles (transfersomes) with dissolving microneedles to significantly enhance transdermal drug delivery, surpassing the performance of either system used individually.

#### In-vivo pharmacokinetics study

**Fig. 12:**
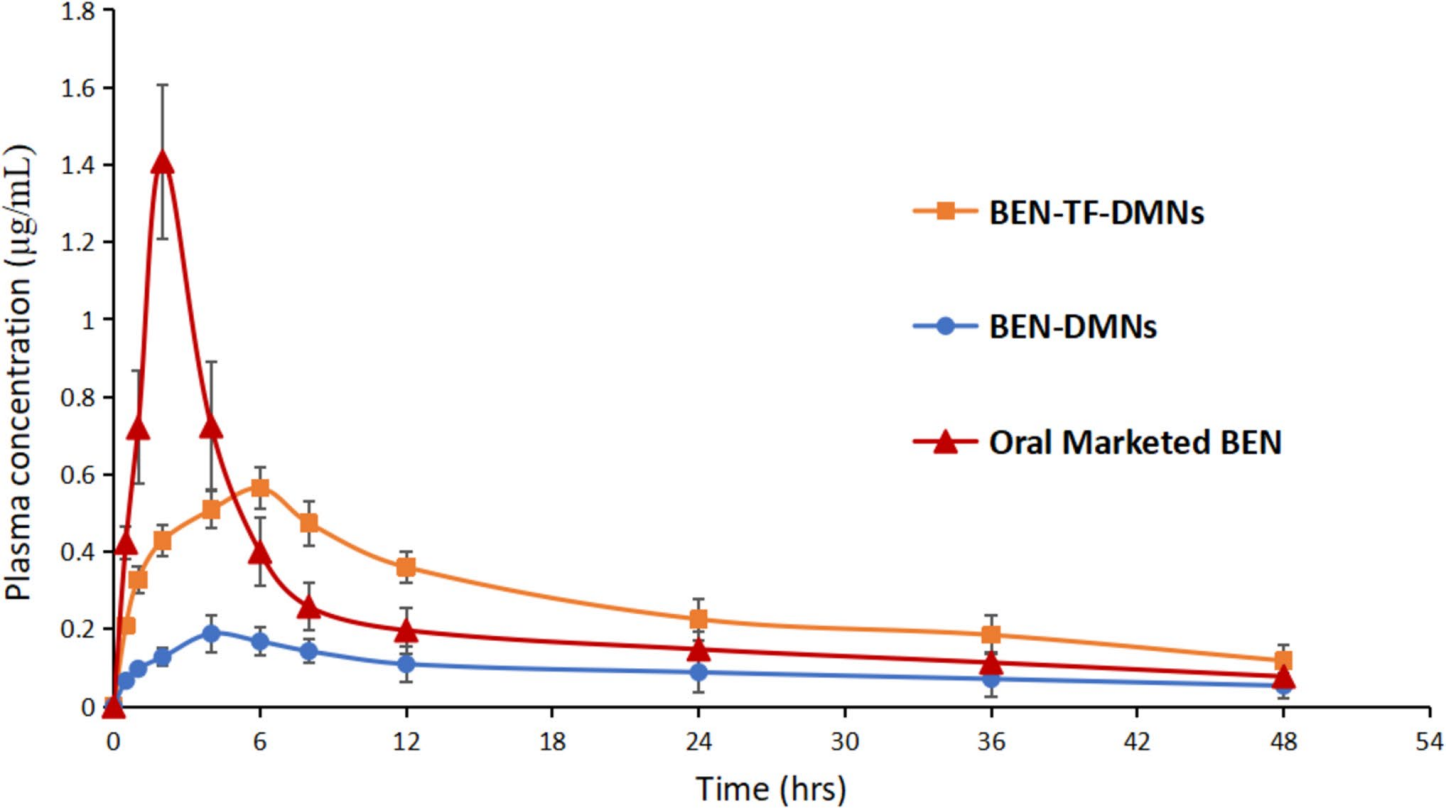
Plasma concentration–time profile of BEN after transdermal application of BEN-DMNs, and BEN-TF-DMNs and oral administration of marketed BEN tablet to rats, (mean ± SD), *n* = 3

To evaluate the bioavailability of BEN, pharmacokinetic profiles like C_max_, T_max_, AUC_0-48_, AUC_0-inf_, K_el_, and t_1/2_ were calculated using a non-compartmental model. The orally marketed tablets of BEN (oral control) and BEN-DMNs (transdermal control) were compared to the bioavailability of BEN-TF-DMNs formulation. Figure 12 displays the plasma concentration of BEN in Sprague–Dawley rats after all transdermal formulations and oral tablets have been applied. Table 4 lists the pharmacokinetic parameters for each formulation.

The C_max_ for the BEN-TF-DMNs after the application was found to be 0.564 ± 0.055 μg/ml and the T_max_ was 6 h. The oral-marketed BEN (oral control) showed a plasma concentration of C_max_ of 1.409 ± 0.199 μg/ml and T_max_ 2 h, while BEN-DMNs (transdermal control) showed a plasma concentration of C_max_ of 0.188 ± 0.049 μg/ml and T_max_ 4 h. The difference between C_max_ and T_max_ of BEN-TF-DMNs, and oral-marketed BEN was significant (*p*-value < 0.05). Moreover, oral-marketed tablets of BEN compared with the transdermal route showed a faster elimination rate (0.050 ± 0.01 h^−1^) and shorter half-life (13.966 ± 1.969 h) with a *p*-value < 0.05. This confirms that currently marketed oral tablets must be administered frequently. In contrast, both the BEN-DMNs and BEN-TF-DMNs exhibited slower elimination rates and longer half-lifes compared to the oral-marketed BEN tablets. This suggests that the transdermal route offers a sustained release profile, enabling prolonged maintenance of plasma BEN levels.

Additionally, the AUC values and relative bioavailability for BEN-TF-DMNs were significantly higher than those of BEN-DMNs and oral-marketed BEN tablets as illustrated in Table 5. These findings highlight the limitations of oral administration, particularly due to the hepatic first-pass effect, which can substantially reduce the systemic availability of active compounds and necessitate higher dosing to achieve therapeutic efficacy [105]. Therefore, it can be concluded that the integration of transfersomes into DMNs markedly enhances the relative bioavailability of BEN by approximately 1.35-fold compared to oral tablets, and 2.49-fold compared to BEN-DMNs.

**Table 5:** Pharmacokinetic parameters of BEN-DMNs, BEN-TF-DMNs and oral administration of marketed BEN tablet to rats, (mean ± SD), *n* = 3

Formulation code	Pharmacokinetic parameters					
	C_max_ (μg/mL)	T_max_ (hr)	AUC_0-t_ (μg/hr/mL)	AUC_0-∞_ (μg/hr/mL)	K_el_ (hr^−1^)	T_1/2_ (hr)
BEN-TF-DMNs	0.564 ± 0.055	6.00 ± 0.0	13.079 ± 1.274	16.487 ± 2.217	0.035 ± 0.01	19.758 ± 1.241
BEN-DMNs	0.188 ± 0.049	4.00 ± 0.0	4.552 ± 0.609	6.649 ± 0.392	0.026 ± 0.01	27.093 ± 4.959
Oral-Marketed BEN	1.409 ± 0.199	2.00 ± 0.0	11.061 ± 1.742	12.653 ± 2.347	0.050 ± 0.01	13.966 ± 1.969

## Conclusion

This study successfully demonstrated the development of a novel transdermal delivery system by integrating benidipine-loaded transfersomes into dissolving microneedles. The transfersomal formulation, optimised using a statistical design approach, exhibited desirable physicochemical characteristics, including nanoscale vesicle size, high entrapment efficiency, and enhanced skin permeability. When incorporated into dissolving microneedles, the resulting BEN-TF-DMNs exhibited rapid dissolution, sufficient mechanical integrity, and efficient skin insertion. Ex vivo and in vivo findings confirmed that this hybrid system significantly improved benidipine’s transdermal permeation and bioavailability compared to both the conventional oral formulation and individual component systems (BEN-TF or BEN-DMNs alone). The synergistic effect of combining chemical and physical enhancement strategies offers a promising, minimally invasive platform for the effective systemic delivery of benidipine and potentially other drugs with limited oral bioavailability. Further investigations in larger animal models and clinical studies are warranted to validate its translational potential.

## Data Availability

The original contributions presented in the study are included in the article; further inquiries can be directed to the corresponding author.
